# Nonalcoholic Fatty Liver Disease (NAFLD) as Model of Gut–Liver Axis Interaction: From Pathophysiology to Potential Target of Treatment for Personalized Therapy

**DOI:** 10.3390/ijms22126485

**Published:** 2021-06-17

**Authors:** Francesca Fianchi, Antonio Liguori, Antonio Gasbarrini, Antonio Grieco, Luca Miele

**Affiliations:** 1 Dipartimento di Scienze Mediche e Chirurgiche, Fondazione Policlinico Universitario A. Gemelli IRCCS, 00168 Rome, Italy; francesca.fianchi@gmail.com (F.F.); lig.antonio91@gmail.com (A.L.); Antonio.gasbarrini@unicatt.it (A.G.); antonio.grieco@unicatt.it (A.G.); 2 Dipartimento Universitario di Medicina e Chirurgia Traslazionale, Università Cattolica del S. Cuore, 00168 Rome, Italy

**Keywords:** microbiota, NAFLD, MAFLD, NASH, hepatocarcinoma

## Abstract

Nonalcoholic fatty liver disease (NAFLD) is the leading cause of liver disease worldwide, affecting both adults and children and will result, in the near future, as the leading cause of end-stage liver disease. Indeed, its prevalence is rapidly increasing, and NAFLD is becoming a major public health concern. For this reason, great efforts are needed to identify its pathogenetic factors and new therapeutic approaches. In the past decade, enormous advances understanding the gut–liver axis―the complex network of cross-talking between the gut, microbiome and liver through the portal circulation―have elucidated its role as one of the main actors in the pathogenesis of NAFLD. Indeed, evidence shows that gut microbiota is involved in the development and progression of liver steatosis, inflammation and fibrosis seen in the context of NAFLD, as well as in the process of hepatocarcinogenesis. As a result, gut microbiota is currently emerging as a non-invasive biomarker for the diagnosis of disease and for the assessment of its severity. Additionally, to its enormous diagnostic potential, gut microbiota is currently studied as a therapeutic target in NAFLD: several different approaches targeting the gut homeostasis such as antibiotics, prebiotics, probiotics, symbiotics, adsorbents, bariatric surgery and fecal microbiota transplantation are emerging as promising therapeutic options.

## 1. Introduction

The human gut microbiota comprises the trillions of microorganisms from 500 to 1000 different species that reside in our gastrointestinal (GI) tract, of which bacteria constitute the vast majority [1,2]. This large community of microbes establishes a symbiotic relationship with the host and is able to perform various functions that significantly influence its physiology and pathology. Indeed, the gut microbiota has recognized to play many important roles in the mammalian body such as dietary nutrient metabolism and energy extraction, immune system education and tolerance development and the prevention of pathogen colonization [3,4,5]. For this reason, disturbance of its homeostasis may be involved in the development of several different diseases.

Studies of the human microbiome revealed that the population of microbes inhabiting the gut have an interpersonal variation and temporal fluctuations in composition even in healthy individuals; much of this diversity remains unexplained although diet, environment, host genetics, antibiotics, lifestyle factors and early microbial exposure have all been implicated [6,7].

In the past decade a close relationship between gut microbiota homeostasis and liver diseases has been described [8,9,10]. In fact, the liver receives 75% of its blood supply from the intestinal portal circulation and provides first-pass metabolism for the GI luminal contents: These include dietary nutrients as well as toxins and xenobiotics that translocate across the intestinal epithelium [11]. Several different studies have described a beneficial role of the commensal microbiota in maintaining liver homeostasis and preventing liver fibrosis [12]. As such, a disturbed imbalance of the endogenous microbiota, otherwise known as dysbiosis, has been associated with a range of chronic liver conditions such as NAFLD, nonalcoholic steatohepatitis (NASH), alcoholic liver disease (ALD) [13] as well as cirrhosis and its complications (hepatic encephalopathy (HE), HCC) [14,15,16,17].

On the other hand, it is well known that liver diseases can alter the gut microbiota, in a mutual relationship in which the two systems are able to influence each other. For this reason, authors have started to talk about the existence of a “gut–liver axis” [18,19].

## 2. Gut Microbiome and NAFLD

NAFLD is estimated to affect up to one-third of the adult population in many developed and developing countries, and its global incidence is continuously rising along with the global rise of obesity and metabolic syndrome [20].

Indeed, NAFLD should be considered as a spectrum of chronic liver diseases that ranges from simple steatosis to NASH and cirrhosis [21]. NAFLD is considered to be the hepatic manifestation of metabolic syndrome. For this reason, a group of experts has recently suggested a change in nomenclature for these conditions that could better reflect the pathogenesis and the heterogeneity of patients: metabolic (dysfunction) associated fatty liver disease “MAFLD” has been proposed [22].

The pathophysiology is multifactorial, involving genetic and epigenetic factors, insulin resistance, hormones secreted from the adipose tissue and nutritional factors [23]. The result of this multiple-hit pathogenesis is a lipid accumulation in the hepatocytes with consequent oxidative stress, lipid peroxidation, adipokine signaling and pro-inflammatory cytokine expression [24]. In addition to the well-known risk factors for the disease (genetics, Western diet and sedentary lifestyles), the important role played by the gut microbiota and its metabolites has increasingly emerged [25]. In fact, a growing body of evidence suggests that the gut microbiota is able to modulate the gut–liver axis [26,27,28].

Both animal and human studies have identified compositional changes in gut microbiota in association with NAFLD-spectrum diseases, although some contrasting evidence has emerged. Indeed, hepatic steatosis has been associated with a reduced diversity of the microbiota population inhabiting the gut. At the phylum level, an increase in Firmicutes and a reduction in Bacteroidetes is commonly described in NAFLD, although diverging results have been described at the class, order, family and genus levels [29].

An elegant study from Raman et al. compared 30 NAFLD patients and 30 healthy controls, finding increased Lactobacillaceae, Lachnospiraceae and Veillonellaceae, but decreased Ruminococcaceae in NAFLD patients [30]. Mouzaki et al. found that patients with NASH had a lower percentage of Bacteroidetes compared to both patient with NAFLD and healthy controls [31]. Zhu et al. demonstrated that children with biopsy-proven NASH had significant difference in gut microbiota composition compared to children with obesity alone: in particular, authors found increased *Bacteroidetes in* NASH, in contrast to results by Mouzaki [32].

Examining all the data emerged from human studies, certain taxonomic changes have been observed in association with NAFLD but several controversies regarding the microbiome profile in this population are still existing—likely due to differences in experimental design—and no consistent microbiota signature has been identified yet [33].

On the other hand, changes in bacterial taxonomy might not be as important as changes in bacterial genes (metagenomics and metatranscriptomics) in the pathogenesis of NAFLD: In fact, the complex interactions between the enteric microbiome and the host are often mediated by metabolites. In a recent study conducted by Qian et al. in mice, it was demonstrated, for example, that the transcriptional landscape changes dramatically in mice with NASH when compared to mice with isolated steatosis. Plasma lipidome analysis demonstrated a very clear difference between these two groups of mice, which was partially recapitulated in serum of patients with isolated steatosis and NASH [34].

In detail, NAFLD and its severity seem to be associated with a greater abundance of genes encoding inflammatory bacterial products [35].

A great challenge at this point was to establish whether dysbiosis could play a causal role or rather represent an epiphenomenon of the disease. To this end, several studies have investigated the potential causal relationship between microbiota and NAFLD: Animal studies in which the gut microbiota is manipulated and observational studies in patients with NAFLD have provided considerable evidence that dysbiosis contributes to the pathogenesis of NAFLD. A crucial murine investigation conducted by Le Roy et al. demonstrated that the risk of developing NAFLD is transmissible by fecal transplant to recipient mice: Indeed, gut microbiota transplantation from a mouse that had previously showed a metabolic response to high-fat diet to germ-free mice produced hyperglycemia, hyperinsulinemia and hepatic steatosis. On the other hand, mice accepting microbiota from a donor mouse that had not developed a metabolic response to high-fat diet, remained normoglycemic and did not develop steatosis. Interestingly, these results were independent from weight gain, which had been comparable between the two donors. The characterization of the transplanted microbiotas revealed significant differences at the phyla, genera and species levels, highlighting the role of dysbiosis [36]. A human study by Vrieze et al. demonstrated that transfer of intestinal microbiota from lean donors could increase insulin sensitivity in individuals with metabolic syndrome, along with increased levels of butyrate-producing intestinal microbiota [37].

Interestingly, gut dysbiosis seems to be implicated not only in the development of NAFLD but also in the severity of disease and its progression to NASH and, eventually, cirrhosis [38]. Boursier et al. examined the gut microbiota taxonomic composition of patients with biopsy proven NAFLD and different degrees of fibrosis: The results showed how *Bacteroidetes* abundance was significantly higher in NASH, whereas *Prevotella* abundance was lower; *Ruminococcus* abundance was significantly higher in F ≥ 2 patients [39]. As a result, these two genera of bacteria seem independently correlated to the severity of disease. These results were compliant with the previously cited study from Mouzaky et al. [31]. A case series by Bastian et al. highlighted how gut microbiota profiles in NAFLD seem to affect disease progression in terms of liver fibrosis, evaluated with transient elastography [40]. A recent cross-sectional study conducted in children described how fecal microbiomes of patients with NAFLD have lower α-diversity than those of control children, and among them, children with NASH have the lowest α-diversity. When considering the microbial populations, a high abundance of Prevotella copri was associated with more severe fibrosis [35].

**Figure 1:**
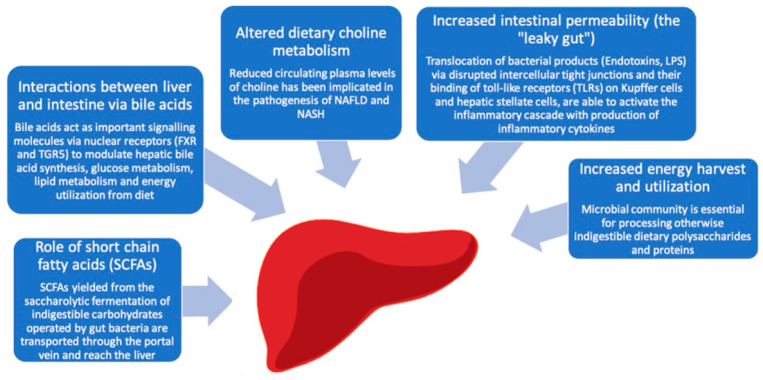
Gut microbiota related mechanisms implicated in the pathogenesis of NAFLD/NASH. Intestinal microbiota can contribute to the development of hepatic steatosis through a variety of mechanisms, including an increased intestinal permeability, effects on appetite regulation and energy extraction from the diet and modulation of bile acids signaling.

Several different mechanisms have proposed to explain the role of the gut microbiota in the pathogenesis of NAFLD, including a dysbiosis-induced increased intestinal permeability (the “leaky gut”), an increased dietary energy harvest, the regulation of choline metabolism, the production of short chain fatty acids (SCFAs) and the bile acids metabolism [Figure 1].

### 2.1. The “Leaky Gut”

Dysbiosis has been associated with a dysregulation of gut endothelial barrier function, with enhanced intestinal permeability to microbes and/or microbial products (endotoxins, lipopolysaccharide [LPS], peptidoglycan) that enter the portal circulation with an increased hepatic exposure to injurious substances, potentially leading to hepatica inflammation and fibrosis [41,42]. Indeed, translocated bacteria or their products are important mediators of liver inflammation and fibrosis by binding to receptors of the innate immune system on the liver cells [43]. The abundance of this microbial product in the portal circulation elicits host immunological responses through the activation of toll-like receptors (TLRs) on Kupffer cells and hepatic stellate cells, with consequent activation of the inflammatory cascade and production of inflammatory cytokines such as tumor necrosis factor (TNF) α, interleukin (IL)-1β and interferons [44].

Among the TRLs family, TLR2, TRL4, TRL5 and TRL9 are implicated in the pathogenesis of NAFLD [45]. TLR4 is involved in NAFLD development through its binding with LPS and activation of NF-kB pathway [46,47], as demonstrated by the fact that mutant mice with a disruption of the LPS-TLR4 signaling pathway (TLR4 mutant mice) are resistant to liver injury and fibrosis, as well as being protected against diet-induced obesity and insulin resistance [48,49,50]. Consistent with histological findings in the liver, the expression of proinflammatory cytokines is suppressed in TLR4 mutant mice, even in the presence of equivalent LPS levels. In another study, TLR9 promoted steatohepatitis by the induction of (IL)–1β in mice [51]. Indeed, even though gut microbiota represents a source of TLR ligands, in healthy livers there is a high tolerance to the TRL ligand because hepatic cells express minimal TLRs. In contrast, TLR signaling is activated and downstream molecules are increased in NAFLD due to a disrupted immunological tolerance.

This inflammatory milieu represents the first line of defense against the invading pathogens; however, sustained elevation of these cytokines injures the host. Indeed, the presence of portal inflammation in NAFLD may contribute directly to fibrogenesis and is strongly related to disease severity [52].

The molecular mechanisms underlying the increased gut permeability are described in detail in NAFLD. In particular, the translocation occurs via disrupted intercellular tight junctions [53]. In murine models, it has been demonstrated that the loss of junctional adhesion molecule A induced by a high-fat diet promotes severe steatohepatitis [54]. In a colitis model induced by dextran sulfate sodium (DSS) in mice, bacterial endotoxins have shown to promote NASH progression [55]. In human models, Miele et al. showed enhanced intestinal permeability related to a decreased expression of zonula occludens-1 (ZO-1), a protein involved in mucosal tight junctions [56].

Indeed, the proven association between gut barrier dysfunction and NAFLD development does not necessarily imply causation, thus it is not clear whether increased intestinal permeability should be considered as a cause or rather a consequence of NAFLD, and further studies are needed to address this major issue. On the other hand, some evidence suggest that early-phase hepatic injury and inflammation are able to contribute to intestinal permeability in a vicious circle. In a meta-analysis of five studies recruiting 128 NAFLD patients, 39.1% of NAFLD patients showed an increased intestinal permeability compared with 6.8% of healthy controls, and the rate of patients with gut barrier dysfunction arose as high as 49.2% when the NASH subgroup was analyzed. In order to further address the underlying mechanism of action, the authors studied changes in intestinal permeability in a diet-induced (methionine-and-choline-deficient; MCD) murine model of NASH: They showed that liver injury is induced early in the course of the MCD diet, before any change in intestinal permeability occurs, suggesting that early changes in liver physiology may affect intestinal homeostasis and contribute to the intestinal permeability [57]. Accordingly, in another study from Giorgio et al., intestinal permeability, as measured through lactulose–mannitol test, appeared to be higher in children with NASH than in children with NAFLD [58].

### 2.2. Increased Dietary Energy Harvest and Utilization by the Microbiota

The gut microbiota plays a key role in regulating the amount of energy extracted from dietary food: indeed, the microbial community is essential for processing otherwise indigestible dietary polysaccharides and proteins by providing a variety of enzymes required for their metabolism [59]. Microorganisms harboring the colon can ferment starch, unabsorbed sugars, cellulosic and non-cellulosic polysaccharides and mucins into short-chain fatty acids (SCFAs) and gases such as carbon dioxide (CO_2_), methane (CH4) and hydrogen (H2) [60,61]. The result of these metabolic reactions is an increased absorption of monosaccharides from the gut lumen.

A close relationship between intestinal microbiota composition and obesity has also been described, both in experimental and in human models. Indeed, the gut microbiota community has been shown to differ between obese mice and their lean littermates, as well as between obese and lean human volunteers [62]; in particular, obesity has been associated with increased Firmicutes rate and decreased Bacteroidetes rate [63]. Consequent metagenomic and biochemical analyses explained how these changes may influence the metabolic potential of the gut microbiota: The obese microbiome, in fact, showed an increased capacity to harvest energy from the diet, with consequent elevated caloric absorption and induction of de novo hepatic lipogenesis [64]. In addition, these enteric bacteria are able to suppress the synthesis of fasting-induced adipocyte factor (Fiaf; also known as angiopoietin-like 4), resulting in the increased activity of lipoprotein lipase (LPL) and the consequent accumulation of triglycerides in the liver. This process provides a direct link between the intestinal microbiota and fat deposition in the liver.

Interestingly, this trait is transmissible, as demonstrated by the fact that colonization of germ-free mice with an ‘obese microbiota’ results in a significantly greater increase in total body fat than colonization with a ‘lean microbiota’ [65].

### 2.3. Altered Dietary Choline Metabolism by the Microbiota

Choline is another important metabolite that is involved in the pathogenesis of NAFLD and NASH: choline deficiency in the diet has been linked to liver diseases for a long time and choline-deficient diets have historically been used to create rodent models of NASH [66].

Subsequently it has been described how choline deficiency could occur under pathophysiologic conditions, and an association with the gastrointestinal microbiome composition has been described [67]. Indeed, it has been demonstrated that high-fat diets in mice are conducive to the development of an intestinal microbiota with the ability to convert dietary choline into methylamines, with consequently low circulating levels of plasma phosphatidylcholine and high urinary excretion of methylamines (dimethylamine, trimethylamine and trimethylamine-N-oxide) [68]. As a result, the reduced circulating plasma levels of choline mimic the effect of choline-deficient diets, contributing to the development of NAFLD. This microbiota-related reduced choline bioavailability may result in the inability to synthesize phosphatidylcholine, which is necessary for the assembly and secretion of very-low-density lipoprotein (VLDL) [69], therefore resulting in triglyceride accumulation in hepatocytes.

Interestingly, microbiota-induced choline deficiency has also been associated with atherosclerosis and cardiovascular disease [70,71].

### 2.4. The Role of SCFAs

There is a growing interest in the pathogenesis of NAFLD by short chain fatty acids (SCFAs) (i.e., propionate, acetate, butyrate) yielded from the saccharolytic fermentation of indigestible carbohydrates operated by gut bacteria [72]. In fact, although most SCFAs are used in the gut as source of energy, some amount is transported through the portal vein and reaches the liver. In particular, while butyrate is the preferred nutrient for epithelial colonic cells, substantial amounts of propionate are absorbed into the bloodstream and transported to the liver, where it serves as a substrate for gluconeogenesis and cholesterol synthesis. Acetate is the principal SCFA in the blood and is an important energy source to peripheral tissues, including the liver, where it is used for lipogenesis and cholesterol synthesis [73].

SCFAs have been widely related to obesity [74,75], through several different mechanisms such as appetite regulation, energy harvesting and expenditure, insulin resistance, adipose tissue metabolism and diabetes development [76,77]. SCFAs exert their metabolic activity through their binding to G-protein-coupled receptors: they have been demonstrated to represent potent signaling molecules when binding to receptors such as GPR41, GPR43 and GPR109A. One of the mechanisms by which SCFAs affect fat accumulation both in the liver and in the adipose tissue is regulation of insulin sensitivity via GPR43 [78], a metabolite-sensing receptor that heavily influence inflammatory responses through the regulation of the inflammasome [79,80].

Once establishing their role in the development of obesity, SCFAs have been observed to be involved in NAFLD, even though their exact role in the pathogenesis of the disease remains unclear. Rau et al. found higher fecal SCFAs concentrations and a domination of SCFAs-producing bacteria in NAFLD patients when compared to healthy controls, and SCFAs concentrations were associated with immunological features of NAFLD disease progression toward NASH [81].

### 2.5. Interactions between Liver and Intestine via Bile Acids

Bile acids (BA) are endogenous molecules well known for their effects on cholesterol homeostasis and lipid digestion. In addition to facilitating fat absorption, bile acids also act as signaling molecules via nuclear and plasma membrane receptors, of which there are the farnesoid X receptor (FXR) and the Takeda G-protein coupled receptor clone 5 (TGR5). With the identification of these molecular pathways, further roles have been elucidated for bile acids including glucose and lipid metabolism as well as having a role in atherosclerosis, insulin sensitization, inflammation and liver fibrosis development [82,83,84]. Indeed, by binding to the FXR and TGR5, bile acids are able to increase insulin sensitivity and decrease hepatic gluconeogenesis and circulating triglycerides.

Due to the aforementioned role in metabolism, BA are involved in the pathogenesis of several metabolic disease, including obesity, type 2 diabetes, dyslipidemia and NAFLD [85].

A key role of gut bacteria in the metabolism of bile acids has been well recognized and widely described, influencing BA production and composition as well as their conversion into secondary bile acids and their enterohepatic circulation, in an FXR-dependent manner [86]. Conversely, bile acids can shape the gut microbiota composition both directly via antibacterial activity and indirectly via FXR, in a well-balanced equilibrium [87,88,89]. In detail, the gut microbiota regulates expression of several enzymes involved in bile acid synthesis, including CYP7A1, CYP7B1 and CYP27A1. After their synthesis from cholesterol, the liver conjugates bile acids with glycine or taurine: The synthesis of taurine as well as bile acid acyl-CoA-synthetase, which is the first of two enzymes required for bile acid conjugation, is also under microbial regulation. In addition, the gut microbiota may not only regulate the synthesis and conjugation of bile acids, but also their intestinal uptake through the regulation of apical sodium dependent bile acid transporter (ASBT), the main receptor responsible for the enterohepatic circulation of BA [90,91]. Finally, the gut microbiota is also responsible for their metabolism: Indeed, microbial deconjugation (i.e., removal of the glycine or taurine conjugate) that prevents active reuptake from the small intestine is carried out by intestinal bacteria [92].

NAFLD-related dysbiosis has been associated with an altered homeostasis of the bile acids, which in turn increases the risk of hepatic injury. Indeed, dysbiosis may alter the amount and composition of the bile acid pool, resulting in the reduced signaling of bile acid receptors such as FXR and TGR5.

Mouzaki et al. observed a higher synthesis of bile acids in patients with NAFLD compared to healthy controls, as well as a higher primary to secondary bile acids ratio [93]. Jiao et al. confirmed an elevated bile acids production in NAFLD; they also described how NAFLD-associated gut microbiota had increased abundance in Escherichia and Bilophila, both able to metabolize taurine and glycine, with increased production of secondary bile acids. However, despite the elevated production of bile acids, a suppressed hepatic bile acid signaling was described, highlighting the role of the membrane receptors in the pathogenesis of NAFLD [94]. Yang et al. found that patients with NAFLD had lower expressions of FXR in the liver and elevated levels of triglyceride synthesis [95], suggesting that decreased FXR activity is an important factor in the pathogenesis of fatty liver.

The molecular mechanisms by which FXR protects the liver from developing NAFLD are related not only to its role in regulating lipid metabolism but also in suppressing liver inflammation cascade: In particular, FXR exerts its anti-inflammatory effects via antagonizing NF-κB function and inducing acute phase response proteins [96,97].

### 2.6. Dietary Modification of Microbiota

It has been widely reported how dietary intake represents one of the main determinants of the gut microbiome composition, and diet-induced modifications of the microbiome are able to produce dramatic changes in metabolism of the host [98,99]. Indeed, the diet and the intestinal milieu interact in a complex way with the bacterial population in the gut: For example, dietary intake of fibers lead to high amounts of SCFAs and lowers the pH in the colon, which in turn affects the composition of the colonic microbiota and thereby the SCFA production. Evidence also suggests that certain food components, which are able to influence the severity of NAFLD, do so at least in part by changing the gut microbiota. For example, one study by Zeng et al. found that a high-fat diet (HFD) in mice led to increased hepatic lipid accumulation and inflammatory cell infiltration that were associated with increased abundance of Lactobacillus acidophilus [100]. It is believed that the Western diet, with its reduced content of indigestible carbohydrates and an increased fat and protein amount, may alter the pool of gut microbial metabolites shifting from saccharolytic to proteolytic fermentation [101,102]. Therefore, there is a larger amount of protein-derived metabolites such as hydrogen sulfide, ammonia and phenolic compounds, which have shown to exert detrimental effects on gut permeability and might indirectly contribute to NAFLD facilitating toxic molecules drainage into the portal blood [103,104]. By contrast, indole, specifically derived from the microbial metabolism of L-tryptophan, has shown to decrease gut inflammation as well as preventing gut barrier dysfunction [105]. Of note, in a study conducted in mice, oral administration of indole shaped LPS-induced inflammation through downregulation of the NF-κB pathway in the liver [106].

## 3. NAFLD, Microbiota and Hepatocellular Carcinoma (HCC)

Metabolic liver disease dramatically increases the prevalence of hepatocellular carcinoma (HCC) [107,108], as demonstrated by the fact that NAFLD is the most rapidly growing indication for liver transplantation in patients with HCC [109]. Of note, nonalcoholic steatohepatitis can promote hepatocarcinogenesis even in the absence of cirrhosis [110]; consequently, NAFLD-HCC can escape tumor surveillance, and is more often diagnosed at a later tumor stage [111].

The pathogenesis of NAFLD-associated HCC is a complex landscape, involving several different mechanisms such as immune and inflammatory responses, DNA damage, oxidative stress and autophagy [112,113,114,115]. The gut microbiome is involved in most of these mechanisms, being one of the main protagonists of hepatocarcinogenesis [116]. For example, endotoxin accumulation, which is mediated by an altered gut microbiota, prevents carcinogen-induced apoptosis and promotes liver tumorigenesis through the modulation of the immune response, as demonstrated by the fact that circulating levels of LPS are elevated in animal models of HCC and prolonged treatment with low-dose LPS significantly increases HCC development [117]. Indeed, the interaction between LPS and toll-like receptor 4 (TLR4) is crucial in the initiation and promotion of hepatocarcinogenesis through inflammation, chronic liver injury and fibrosis [118]. Conversely, the reduction of LPS trough intestinal decontamination with antibiotics, gut microbiota modulation by probiotics or genetic ablation of its receptor toll-like receptor 4 (TLR4) in mice are protective against tumor growth, representing potential targets for HCC prevention [119].

Another mechanism by which gut bacteria may contribute to hepatocarcinogenesis is through the modulation of bile acid metabolism [120]: Yamada et al., in an experimental model of NASH, suggested that the critical role of the gut microbiota in the conversion of primary to secondary bile acids is involved in HCC development [121].

Moving from this pre-clinical evidence, a study conducted in humans described how HCC development in NAFLD-related cirrhosis is associated with specific gut microbiota profiles and with systemic inflammation. Indeed, patients showed increased levels of fecal calprotectin as well as higher levels of inflammatory cytokines when compared to patients without HCC. When considering the gut microbiota profile, Bacteroides and Ruminococcaceae were increased in the HCC group, while Bifidobacterium were reduced. These results suggest that the gut microbiota composition is significantly related to systemic inflammation, and that it may be involved in hepatocarcinogenesis [122].

## 4. Diagnostic Potential of Gut Microbiota

As extensively reported gut microbiota is involved not only in NAFLD development, but also in its progression and severity, and for this reason it may be considered as a promising non-invasive marker of disease. In assessing whether the severity of NAFLD is associated with gut dysbiosis, the above-mentioned study from Boursier et al. identified Bacteroides as independently associated with NASH and Ruminococcus with significant fibrosis [39]. In another study, adult patients with NASH had a significantly higher percentage of Clostridium coccoides than patients with NAFLD [31]. The analysis conducted from Bastian et al. found significant differences in the composition of gut microbiota in NAFLD patients based on the stages of liver fibrosis, suggesting, for example, that the proportion of Bacteroides can represent a marker of significant liver fibrosis [40].

Given the association between specific microbial populations and NASH, as long as liver fibrosis, a strong rationale exists for the development of a panel of gut-microbiome-derived biomarkers that could predict the presence of advanced fibrosis. Moving from this evidence, a study conducted by Loomba et al. provides the preliminary evidence for a fecal-microbiome-derived metagenomic signature to detect advanced fibrosis in NAFLD [123]. Thus, gut microbiota composition analysis adds information to the classical predictors of NAFLD severity and for this reason can represent a main protagonist in the great challenge of noninvasive assessment of liver disease severity.

## 5. Therapeutic Potential of Gut Microbiota

There is currently no medical treatment or drug approved for NAFLD/NASH other than dietary and lifestyle recommendations, although some promising trials are ongoing. In the recent years, modulation of the gut microbiota has been proposed as a new promising therapeutic approach in NAFLD, and the effectiveness of therapies such as antibiotics, probiotics, prebiotics, synbiotics, absorbents, anti-inflammatory drugs and fecal microbiota transplantation have been assessed in several studies [124].

### 5.1. Antibiotics

Antibiotics administration, with the aim of reducing the enteric burden of bacteria and the translocation of microbial components has been extensively studied in NAFLD [125]. In particular, Rifaximin, a non-absorbable antibiotic acting on Gram-negative bacteria, has been shown to exert beneficial effects in patients with NAFLD/NASH, due to its role in lowering endotoxaemia and proinflammatory cytokine production [126,127,128]. In addition, Rifaximin seems to exert an “eubiotic role”, being capable of inducing the overgrowth of beneficial bacteria such as Bifidobacterium, Faecalibacterium and Lactobacillus and so exerting an anti-inflammatory effect [129].

### 5.2. Prebiotics

Prebiotics are indigestible substrates that promote the growth of beneficial bacteria in the gastrointestinal tract that, in preclinical studies, have shown a role in improving the biochemical and histologic markers of NAFLD [130,131]. Despite their safety profile and low cost, only limited studies on the efficacy of prebiotics in NAFLD have been conducted in humans so far [132,133,134,135,136,137], each one with a small sample size, and some of them not even including patients with histologic confirmation of NAFLD. Moreover, in all of these studies, the impact of prebiotics has been assessed using biochemical markers of liver injury or indices of metabolic dysregulation [138]. Larger clinical trials are currently ongoing, having the impact of prebiotics on steatosis and fibrosis determined by histology or imaging studies as their primary outcome.

### 5.3. Probiotics

Probiotics are living microorganisms that in adequate amounts are able to confer a health benefit to the host via competitive colonization and by acidification of the gastrointestinal lumen, with consequent improvement of the mucosal integrity. Several clinical trials have been conducted on humans in order to evaluate the effect of probiotics on NAFLD/NASH [139,140,141,142,143,144,145,146,147,148]: The vast majority of these studies have reported an improvement in serum surrogate markers of disease. Unfortunately, as in the case of prebiotics, only few studies have examined the effect of probiotics on histologic features and, for this reason, their role as a standard therapy in NAFLD/NASH has not been established yet.

Moreover, probiotics seem to have a positive effect in animal models of HCC: in fact, due to their protective effects on intestinal barrier function and their immunomodulatory activity, probiotics are capable of reducing the tumor burden and its inflammatory milieu and can be proposed as a promising weapon in the therapy of NAFLD-related HCC [149,150]. However, human studies are necessary for translating this evidence into clinical practice.

### 5.4. Symbiotics

Symbiotics are a combination of both a prebiotic and a probiotic that currently represent an area of great therapeutic research in NAFLD. Early studies suggested a role in improving both the biochemical and histological features of NAFLD, as well as in ameliorating the overall metabolic profile (in terms of anthropometric indices, lipid profiles and glucose homeostasis parameters) [151,152,153,154,155,156,157]. However, the results of a recent clinical trial (the INSYTE study) were not encouraging in promoting synbiotics as a standard therapy for NAFLD: Indeed, 1 year of administration of a synbiotic combination improved the fecal microbiome but did not reduce liver fat content or markers of liver fibrosis [158].

### 5.5. Metformin

Another possible approach involves drugs with anti-inflammatory effects, with the aim of reducing both the intestinal and systemic inflammation. For example, Metformin has been revealed to act as a potent anti-inflammatory drug through a modulation of the gut microbiota [159,160,161]: In particular, it is able to produce a favorable change in the gut microbiota composition, with an increase in the relative abundance of Bifidobacterium and Akkermansia [162]. Interestingly, Metformin has been associated with a reduced incidence of HCC in preclinical models of NAFLD, probably another effect related to its anti-inflammatory properties [163]. However, currently available human studies of metformin in NAFLD/NASH found no difference from placebo in terms of steatosis, fibrosis, NAFLD activity score or resolution of NASH [164,165,166,167,168].

### 5.6. Bile acid Homeostasis Targeting

As already mentioned, there is a great interplay between intestinal microbiota and bile acids, each one being able to influence the other, and for this reason bile acid homeostasis has been targeted for the treatment of NAFLD. Obeticholic acid (OCA), a steroidal semisynthetic derivative of chenodeoxycholic acid and a potent FXR agonist, has shown to reduce liver fat and fibrosis in animal models of fatty liver disease, and for this reason it has been extensively studied for the treatment of NASH. These beneficial effects of OCA seem related to gut microbiota modulation. Indeed, in cirrhotic rats it has been demonstrated to improve dysbiosis and to reduce bacterial translocation [169]. The FLINT trial assessed the effect of 25 mg of OCA given daily in adults with NASH: the administration of the drug was associated with weight loss and improved liver histology in terms of NAFLD activity score (NAS) and fibrosis [170]. The role of OCA for the treatment of NASH is currently under investigation in a large, randomized, phase III clinical trial (REGENERATE) [171]. Interim analysis of this study showed clinically significant histological improvement after administration of 25 mg Obeticholic acid in patients with NASH [172].

### 5.7. Currently under Investigation: Adsorbents, TLR-4 Signaling, FGF-19 and FGF-21 Signaling

Adsorbents are poorly absorbable, adsorptive materials that are capable of binding gut-derived toxins and bacterial products, thus abrogating their inflow into the liver and systemic circulation with a consequent reduction in endotoxaemia [173]. Preliminary studies in rodents with NAFLD show a marked reduction in steatosis and hepatic inflammation: For example, Yaq-001, a synthetic activated carbon with the ability to selectively absorb intestinal-derived toxins (such as cytokines, hydrophobic bile acid and bacterial products) produced a significant reduction in ALT and hepatic TLR-4 expression in rodents with NAFLD [174]. Currently, clinical trials to assess therapeutic effect of Yaq-001 on humans are under investigation.

The TLR4 signaling, whose role in inflammation and fibrosis has extensively been described above, represents another promising target for the treatment of NAFLD: in particular JKB-121, a weak TLR4 receptor antagonist, has been studied in a phase II trial [175]. However, after 24 weeks of treatment, JKB-121 was found to not be superior to the placebo in reducing serum ALT or liver fat accumulation (measured with magnetic resonance imaging), and further studies are needed to address the role of TLR4 antagonists in the treatment of NAFLD.

As outlined before, bile acids mediate—through their ligand to FXR—the release of the enterokine FGF-19, which plays a key role in liver metabolism of lipid and glycogen. Hence, FGF-19 mimetics have been engineered despite their potentially carcinogenic effects. NGM282 is an engineered variant of human FGF19 that was found to retain the metabolic but not the tumorigenic effect of FGF-19 in preclinical models, and for this reason, it has been studied for the treatment of NASH. A phase II placebo-controlled trail of NGM282 for 12 weeks described a normalization of the hepatic fat content in 26–39% of patients with NASH [176,177]. The use of the drug, however, was associated with an increase in LDL-C levels.

### 5.8. Bariatric Surgery

In a prospective study conducted in 2015, bariatric surgery induced the disappearance of NASH from nearly 85% of patients and reduced the pathologic features of the disease after 1-year of follow-up [178]. This group of researchers analyzed then the same cohort after a 5-year follow-up in order to assess the long-term effects of bariatric surgery in patients with NASH: The resolution of NASH was observed in liver samples from 84% of patients [179].

This extraordinary result seems to once again be related not only to the metabolic effects of the bariatric surgery, but also to a great impact of these procedures on the gut homeostasis. Indeed, it is known, for example, that gastric bypass can favorably affect the gut microbiota by reducing the proportion of firmicutes, by remodeling the bile acid pool and by modulating the secretion of incretins [180,181]. Hence, this surgical procedure is able to affect most of the pathways of the gut–liver-axis.

### 5.9. Fecal Microbiota Transplantation (FMT)

FMT has been used successfully in the treatment of patients with refractory and recurrent Clostridium difficile, and it is increasingly emerging in the clinical practice as a promising therapeutic option for several other diseases, having the potential to restore an ‘‘healthy” microbiome [182]. As for the liver, several different preclinical studies and preliminary experience on humans have outlined the roles of FMT in cirrhosis and in the treatment of its complications [183,184,185]. Bajaj et al. conduced a phase 1 clinical safety trial of FMT in patients with decompensated cirrhosis on standard therapies (lactulose and rifaximin): FMT was well tolerated, and it was able to restore microbial diversity and function [186].

Moving from this evidence, a role of FMT in the treatment of NAFLD is emerging. Indeed, the restoration of a healthy intestinal microbiota has been demonstrated to interfere favorably in the pathogenesis of NAFLD in mouse models [187,188]. One preliminary experience on humans was conducted by Vrieze et al. in 2012: The authors demonstrated that the transfer of intestinal microbiota from lean donors could increase insulin sensitivity in individuals with metabolic syndrome [37].

However, no human studies have established the specific role of FMT in the treatment NAFLD at present. In a recent randomized controlled trial from Craven et al., allogenic FMT in patients with NAFLD did not improve insulin resistance nor did it significantly reduce hepatic fat fraction. However, as an interesting result, FMT showed the potential to reduce small intestinal permeability 6 weeks after the transplant [189].

In a double-blind randomized controlled proof-of-principle study of 2020 allogenic FMT using lean vegan donors in individuals with hepatic steatosis produced a modification of intestinal microbiota composition, which was associated with beneficial changes in plasma metabolites and markers of steatohepatitis [190].

Several other clinical trials exploring the role of FMT in the treatment of NAFLD are currently ongoing.

## 6. Conclusions

NAFLD is nowadays the first cause of liver disease worldwide, affecting both adults and children, and in the near future it will emerge as the leading cause of end-stage liver disease. Indeed, its prevalence is rapidly increasing, and NAFLD is becoming a major public health concern: For this reason, great efforts have made in recent years to identify more the pathogenetic factors in more detail and to find new therapeutic targets. In fact, despite this great epidemiological burden, the pathophysiology of NAFLD is not entirely understood at present.

In the past decade, the enormous advances in understanding the biology of the gut–liver axis have elucidated its role as one of the main actors in the development of NAFLD. Indeed, numerous evidence has implicated the intestinal microbiome in the development and progression of hepatocellular steatosis, inflammation and fibrosis seen in the context of NAFLD, as well as in hepatocarcinogenesis.

Once this close relationship is established, the microbiome has proposed as a useful tool to determine liver disease severity in NAFLD, and the risk of disease progression from NAFLD toward NASH and more severe fibrosis. In other words, gut microbiota is emerging as a potential non-invasive marker of disease-severity in NAFLD. In addition, gut microbiota has a role in the stratification of patient at high risk of developing HCC has proposed as well as a key role in the early diagnosis of hepatocarcinoma. Indeed, despite the great prevalence of NAFLD-related HCC, a structured surveillance program in absence of severe fibrosis remains to be established.

Moving towards its enormous diagnostic potential, the next step was to imagine the gut microbiome as a promising therapeutic target for NAFLD, whose treatment’s options remain limited at present. In particular, preclinical experiences have established the great impact of microbiome-targeted therapies in mice ranging from antibiotics, probiotics, symbiotic to fecal microbiota transplantation (FMT), and studies conducted on humans so far seem to confirm this impact [Table 1]. Large, multicentric clinical trials exploring the metabolic effects of microbiome-targeted therapies in NAFLD and its complications (e.g., HCC) are currently ongoing. 

**Key Points:**

1. NAFLD is the first cause of liver disease worldwide, but reliable non-invasive disease markers and efficacious therapeutic options are currently lacking.
2. Gut microbiota demonstrated its involvement in the pathogenesis of NAFLD spectrum diseases through several different mechanisms.
3. Due to its role in the pathogenesis and progression of NAFLD, gut microbiota may be considered as a potential noninvasive disease biomarker.
4. Modulation of the gut microbiota is currently under study as a promising therapeutic approach in NAFLD and its complications (e.g., HCC).

## Figures and Tables

**Table 1:** Summary of available human studies on treatments targeting the microbiome in NAFLD.

	POPULATION	TREATMENT	STUDY DESIGN	RESULTS
Antibiotics				
Gangarapu et al., 2015 [126]	42 adult patients with biopsy-proven NAFLD (steatosis, *n* = 15; NASH, *n* = 27)	Rifaximin (1200 mg/daily) for 4 weeks	Prospective, open-label, observational cohort study	✓ In the NASH group: mild reduction in BMI and significant reduction ALT, AST, GGT, LDL, ferritin, plasma endotoxin concentration and IL-10 levels. ✓ In the steatosis group: significant reduction in and ferritin. ✓ Not significant effect on serum levels of TLR-4, IL-1, IL-6, IL-12 or TNF-α in either group.
Cobbold et al., 2018 [127]	15 patients with biopsy-proven NASH and elevated aminotransferase values	Rifaximin 400 mg twice daily for 6 weeks	Open-label pilot study	✓ No beneficial effect of rifaximin in patients with NASH.
Abdel-Razik A et al., 2018 [128]	50 patients with biopsy-proven NASH	Rifaximin 1100 mg/day for 6 months (*n* = 25) vs. placebo (*n* = 25)	Multicentric, double-blind, randomized, placebo-controlled study	✓ Significant reduction in homeostatic model assessment, ALT, AST, GGT, endotoxin, TLR-4, IL-6, TNF-α, CK-18 and NAFLD-liver fat score (all *p* < 0.05). ✓ No changes in the lipid profile. ✓ Mild non-statistically significant reduction of BMI.
Prebiotics				
Daubioul et al., 2005 [132]	Patients with biopsy-proven NASH (*n* = 7)	Oligofructose 16 g/day (Raftilose P95^®^) vs. placebo (maltodextrine) for 8 weeks	Randomized double-blind crossover study	✓ Significant reduction of AST (*p* < 0.05 vs. placebo) after 8 weeks, and insulin level after 4 weeks. ✓ Nonsignificant decrease of TG concentrations.
Rocha R et al., 2007 [133]	12 patients with NAFLD	10 g/day of Psyllum plantago husk (Ispaghula husk) for 3 months	Open-label clinical trial	✓ 100% of patients presented reduction in BMI, waist circumference and insulin resistance index. ✓ Reduction of the cholesterol levels in 66.7% of patients. ✓ 75% presented normal liver enzymes (AST, ALT, and GGT).
Ebrahimi-Mameghani et al., 2014 [134]	60 obese adult patients with NAFLD	400 mg/d of vitamin E plus four 300-mg tablets of Chorella vulgaris (ALGOMED) (*n* = 30) vs. placebo (400 mg/d of vitamin E and four placebos/d) (*n* = 30) for 8 weeks	Double-blind, randomized, placebo-controlled clinical trial	✓ Weight, liver enzymes, FBS and lipid profile decreased significantly in both groups (*p* < 0.05), with statistically significant differences in the two groups (*p* = 0.01, *p* = 0.04 and *p* = 0.02, respectively).
Akbarzadeh et al., 2015 [135]	75 overweight or obese adults with NAFLD	10 g of psyllium (Plantago ovata) or 10 g of crushed wheat as placebo for 4 months	Single-blind, placebo-controlled, parallel, randomized clinical trial	✓ Reduction in AST and ALT. ✓ No differences in BMI.
Lambert et al., 2015 [136]	60 adults (BMI ≥ 25) with confirmed NAFLD	16 g/d prebiotic supplemented (*n* = 30) vs. isocaloric placebo (*n* = 30) for 24 weeks	Double blind, placebo-controlled parallel group study	✓ Currently ongoing ✓ ClinicalTrials.gov (NCT02568605).
Javadi et al., 2017 [137]	75 patients with NAFLD divided into four groups (21 patients in the prebiotic group)	Prebiotic group received powder (inulin HP: 10 g/d) and a placebo of probiotics (fat- and lactose-free milk capsules).	Double-blind, placebo-controlled clinical trial	✓ Reduction in AST (*p* = 0.045) and ALT (*p* = 0.041). ✓ No significant changes in the grade of fatty liver.
Probiotics				
Loguercio et al.,2005 [139]	22 patients with biopsy-proven NAFLD	VSL#3a for 3 months	Comparative study	✓ Significant improvement in ALT and AST. ✓ Significant reduction in markers of oxidation (malondialdehyde and 4-hydroynonenal) and in levels of S-nitrosothiol.
Aller et al., 2011 [140]	30 patients with biopsy-proven NAFLD	One tablet per day of probiotic mix (500 million CFU L. bulgaricus and S. thermophiles) or placebo (120 mg of starch) for 3 months	Double-blind, randomized clinical trial	✓ Significant reduction in AST, ALT and GGT levels. ✓ Any effects on glucose, TC, LDL, HDL, TG, insulin, HOMA-IR, IL-6 or TNF-α levels.
Vajro et al., 2011 [141]	20 obese children (age 10.7 ± 2.1 years) with persisting hypertransaminasemia and ultrasound evidence of fatty liver	Oral Lactobacillus Gorbach-Goldin 12 billion CFU/d (*n* = 10), or placebo (*n* = 10) for 8 weeks	Double-blind, placebo-controlled pilot study	✓ Significant decrease in ALT and in peptidoglycan-polysaccharide (average variation vs. placebo *p* = 0.03 for each). ✓ No significant differences in BMI, visceral fat, TNF-α levels or hepatorenal ultrasonographic ratio.
Wong et al., 2013 [142]	20 adults with histology-proven NASH	“Usual care” (*n* = 10) or one sachet of Lepicol b.i.d. (*n* = 10) for 6 months	Randomized controlled trial	✓ Reduction in AST levels and intrahepatic triglycerides (IHTG). ✓ No significant alterations in TG, BMI, ALT, fasting glucose, TC, HDL, LDL, hepatic TG or liver stiffness.
Shavakhi et al., 2013 [143]	64 adults with biopsy-confirmed NASH and persistent elevation of ALT	Two tablets of metformin 500 mg and either probiotic supplement daily (Protexin two tablets per day) (group I, *n* = 31) or placebo (group II, *n* = 32) for 6 months	Randomized clinical trial	✓ Significant reduction in ALT, AST and ultrasound grading of NASH in group I; reduction in AST and ultrasound grading of NASH in group II. ✓ Significant reduction in BMI, TG and TC levels (*p* ≤ 0.02 for all) in both groups.
Nabavi et al., 2014 [144]	72 patients with NAFLD (33 males and 39 females) aged 23 to 63 year old	300 g/d of conventional yogurt (*n* = 36) or yogurt enriched with B lactis Bb12 and L acidophilus La5 (*n* = 36) for 8 weeks	Double-blind, randomized, controlled clinical trial	✓ Significant reduction in ALT, AST, TC and LDL serum levels (*p* < 0.05 for all). ✓ No significant changes in levels of serum glucose, TG, or HDL in each group.
Alisi et al., 2014 [145]	44 obese children with histologically diagnosed NAFLD	VSL#3a (1 sachet/d for patients aged < 10 year or 2 sachets/d for patients aged > 10 year) (*n* = 22) vs. placebo (*n* = 22) for 4 months	Double-blind, randomized clinical trial	✓ Risk of severe steatosis was significantly lower (*p* < 0.001). ✓ Significant reduction in BMI (*p* < 0.001). ✓ Trend towards a reduction in GLP-1. ✓ Mo significant changes were observed for TG, HOMA-IR or ALT levels.
Sepideh et al., 2016 [146]	42 patients with NAFLD	Two capsules/day probiotic or placebo for 8 weeks	Double-blind, randomized clinical trial	✓ Significant decrease in insulin, insulin resistance, TNF-α, and IL-6.
Kobyliak N et al., 2018 [147]	58 type 2 diabetes patients with NAFLD	Multi-strain probiotic “Symbiter” (concentrated biomass of 14 probiotic bacteria genera Bifidobacterium, Lactobacillus, Lactococcus, Propionibacterium) (*n* = 30) vs. placebo (*n* = 28) for 8 weeks	Double-blind, single center, randomized clinical trial	✓ Significant reduction in fatty liver index (FLI), aminotransferase activity, and in the TNF-α and IL-6 levels.
Duseja A et al., 2019 [148]	39 liver biopsy-proven patients with NAFLD	Lifestyle modifications plus an oral multistrain probiotic (675 billion bacteria daily) (*n* = 19) or identical placebo (*n* = 20) for 1 year	Double-blind, randomized clinical trial	✓ In comparison to baseline, hepatocyte ballooning (*p* = 0.036), lobular inflammation (*p* = 0.003) and NAS score (*p* = 0.007) improved significantly in the probiotic group. ✓ Compared with placebo, the NAS score improved significantly in the probiotic group (*p* = 0.004), along with improvements in hepatocyte ballooning (*p* = 0.05) and hepatic fibrosis (*p* = 0.018). ✓ Significant improvement in levels of ALT (*p* = 0.046), leptin (*p* = 0.006), TNF-α (*p* = 0.016) and endotoxins (*p* = 0.017).
Symbiotics				
Malaguarnera et al., 2012 [151]	66 patients with histologically diagnosed NASH	24 weeks of a synbiotic (Bifidobacterium longum plus a prebiotic [fructooligosaccharides]) and lifestyle modification (i.e., diet and exercise) versus lifestyle modification alone	Randomized controlled trial	✓ Significantly lower levels of AST, LDL, TNF-α, C-reactive protein (CRP), HOMA index and serum endotoxin. ✓ Histologic improvement (decreased hepatocellular injury, inflammation and steatosis) (*p* < 0.05).
Eslamparast et al., 2014 [152]	52 patients with NAFLD	Daily 2 synbiotic capsules (each one containing seven probiotic strains and fructooligosaccharides) vs. 2 placebo capsules for 28 weeks	Randomized, double-blind, placebo-controlled clinical trial	✓ Significant reduction in liver enzymes (ALT, AST, GGT), inflammatory markers (CRP, TNF-α and total nuclear factor κ-B p65). ✓ Reduction of 2.99 kPa in the hepatic fibrosis score as determined by transient elastography (*p* < 0.001).
Ferolla et al., 2016 [153]	50 biopsy-proven NASH patients	Lactobacillus reuteri with guar gum and inulin for 3 months and healthy balanced nutritional counseling vs. nutritional counseling alone	Randomized, controlled clinical trial	✓ Symbiotic group presented a reduction in steatosis, lost weight, diminished BMI and waist circumference measurement. ✓ Symbiotic did not improve intestinal permeability or LPS levels.
Asgharian et al., 2016 [154]	80 patients with ultrasound-diagnosed NAFLD	Symbiotic in form of a 500 mg capsule (containing seven species of probiotic bacteria and fructooligosaccharides) or a placebo capsule daily for 8 weeks	Randomized, double-blind, placebo-controlled clinical trial	✓ In the symbiotic group, ultrasound grade decreased significantly compared to baseline (*p* < 0.005). ✓ No significant differences in CRP, ALT or AST levels were observed between groups (adjusted for energy intake).
Asgharian et al., 2017 [155]	80 patients with ultrasound-diagnosed NAFLD	Symbiotic in form of a 500 mg capsule (containing seven species of probiotic bacteria and fructooligosaccharides) or a placebo capsule daily for 8 weeks	Randomized, double-blind, placebo-controlled clinical trial	✓ Significant reduction in weight (*p* = 0.001), body fat (*p* = 0.02) and total cholesterol (*p* = 0.04). ✓ TC and LDL (*p* = 0.04 and *p* = 0.001, respectively) were significantly increased in the placebo group. ✓ TG, HDL and FBS levels remained statistically unchanged in both groups.
Mofidi et al., 2017 [156]	50 lean patients with NAFLD (patients had steatosis and elevated ALT)	Synbiotic supplementation vs. placebo for 28 weeks	Randomized, double-blind, placebo-controlled, clinical trial	✓ Significant reductions in fibrosis and hepatic steatosis. ✓ FBS, TG levels and markers of inflammation.
Scorletti et al.—INSYTE study 2020 [158]	104 participants	Synbiotic (combination of fructooligosaccharides; 4 g/day and Bifidobacterium animalis subsp. lactis BB-12 at a minimum of 10 billion CFU/day) (*n* = 55) or placebo (*n* = 49) for 1 year	Randomized double-blind placebo-controlled trial	✓ Synbiotic administration altered the fecal microbiome but did not reduce liver fat content or markers of liver fibrosis
Metformin				
Haukeland et al., 2009 [164]	48 adult patients with biopsy-confirmed NAFLD	Metformin 2500 mg/d (3000 mg if weight > 90 kg) (*n* = 24) vs. placebo (*n* = 24) for 6 months	Randomized, double-blind, placebo-controlled trial	✓ No significant differences were observed for changes in liver steatosis (assessed either histologically or by CT), NAS-score, liver transaminases or on markers of insulin resistance or inflammation. ✓ In contrast, beneficial effects of metformin were observed on changes in body weight (*p* < 0.001), serum levels of cholesterol (*p* = 0.004), LDL (*p* < 0.001), glucose (*p* = 0.032) and on HbA1c (*p* = 0.020).
Omer et al., 2010 [165]	64 adults with type 2 diabetes or impaired glucose tolerance and biopsy-confirmed NAFLD divided into three groups	Group 1 (*n* = 22) received metformin 1700 mg/day for 12 months	Open-label, randomized, single-center study	✓ No significant decrease in serum transaminase and GGT levels, homeostasis model assessment-insulin resistance and NAS on follow-up biopsy.
Razavizade et al., 2013 [166]	80 adults with NAFLD assessed via ultrasonography and predictive formula divided into two groups	Group 1 received Metformin 1000 mg/d (*n* = 40) for 4 months	Double-blind clinical trial	✓ Significantly decreased serum levels of liver function tests, FBS, TC, LDL, HOMA-IR, LFC and increased serum level of HDL.
Rana et al., 2016 [167]	98 patients with ultrasound diagnosed NAFLD divided into three groups	Group 1 (*n* = 31) received Metformin along with dietary intervention and lifestyle modification for 24 weeks	Randomized trial	✓ Metformin was not effective as add on therapy for NAFLD.
Anushiravani et al., 2019 [168]	150 consecutive patients with NAFLD who were assigned to five groups	Group 2 (*n* = 30) received Metformin 500 mg/day for 3 months	Double-blinded, randomized placebo-controlled trial	✓ No significant benefit of Metformin administration.
Bile acid homeostasis targeting				
Neuschwander-Tetri et al., 2015- FLINT trial [170]	283 patients with non-cirrhotic NASH	Obeticholic acid given orally (25 mg daily) (*n* = 141) or placebo (*n* = 142) for 72 weeks	Multicentre, double-blind, placebo-controlled, parallel group, randomized clinical trial	✓ OCA significantly improved the primary histological outcome (i.e., NAFLD activity score) and reduced liver fibrosis compared with placebo. ✓ Several safety issues, including: (a) pruritus; (b) dyslipidaemia, with increased total and LDL cholesterol and mild, but significant, reductions in HDL; (c) concerns regarding the carcinogenic potential of increased circulating FGF19.
REGENERATE trial [171,172]	~2400 patients with histologic evidence of NASH (including ~2100 patients with stage 2 or 3 liver fibrosis)	Patients are randomized 1:1:1 to receive 10 mg OCA, 25 mg OCA or placebo	Phase 3, double-blind, randomized, long-term, placebo-controlled, multicenter study	✓ Currently ongoing—Interim analysis showed clinically significant histological improvement after Obeticholic acid 25 mg administration in patients with NASH.
Adsorbents				
Safety and tolerability of Yaq-001 in patients with NASH	70 patients with NASH	Standard medical treatment + Yaq-001 (8 g/day) (*n* = 35) vs. standard medical treatment + placebo-control (placebo for 8 g of Yaq-001/day) (*n* = 35) for 48 weeks	Multicentre, randomized, double blinded, placebo-controlled trial	✓ Currently ongoing ✓ clinicaltrials.gov (NCT03962608).
TLR-4 signaling				
Diehl et al., 2018 [175]	65 patients with biopsy-proven NASH with a NAS >/= 4, >/=6% liver fat content (LFC) by MRI and elevated ALT	JKB-121 5 mg, 10 mg or placebo twice daily for 24 weeks	Multicenter, double-blind, randomized, placebo-controlled trial	✓ Not superior to placebo in reducing LFC (determined by MRI) and ALT. ✓ Notable improvement in LFC, ALT and FIB-4 in the placebo group.
FGF-19 signaling				
Harrison et al., 2018 [176]	82 adults with biopsy-confirmed NASH divided into 3 groups	Daily subcutaneous injections with 3 mg NGM282 (*n* = 27), 6 mg NGM282 (*n* = 28) or placebo (*n* = 27) for 12 weeks	Phase II, double-blind, placebo-controlled, randomized controlled trial	✓ Rapid and significant reductions in LFC with an acceptable safety profile. ✓ Increase in LDL levels.
Harrison et al., 2019 [177]	43 patients with biopsy-confirmed NASH	Subcutaneous NGM282 (1 mg, *n* = 24; 3 mg, *n* = 19) once daily for 12 weeks	Open-label study	✓ NGM282 significantly reduced NAS with *p* < 0.001 both in the 1 mg and in the 3 mg group, and fibrosis scores (*p* = 0.035 in the 3 mg group). ✓ Improvement in noninvasive imaging (LFC), serum markers (AST, ALT and fibrogenesis biomarkers) and enhanced liver fibrosis score (ELF).
Bariatric surgery				
Lassailly et al., 2015 [178,179]	180 morbidly obese patients with biopsy-proven NASH	Bariatric surgery (gastric banding; gastric bypass; Roux-en-Y-Gastric-bypass; sleeve gastrectomy)	Prospective study	✓ Bariatric surgery induced the disappearance of NASH from nearly 85% of patients and reduced the pathologic features of the disease after 1 year and after 5 years of follow-up.
Fecal Microbial Transplantation				
Craven et al., 2020 [189]	21 patients with NAFLD	Allogenic (*n* = 15) or autologous (*n* = 6) FMT delivered by using an endoscope to the distal duodenum	Randomized controlled trial	✓ FMT did not improve IR as measured by HOMA-IR or hepatic proton density fat fraction (PDFF) but did have the potential to reduce small intestinal permeability in patients with NAFLD.
Witjes et al., 2020 [190]	21 individuals with hepatic steatosis on ultrasound	Lean vegan donor (allogenic *n* = 10) or own (autologous *n* = 11) FMT; both were performed three times at 8-week intervals	Double-blind, randomized, controlled, proof-of-principle study	✓ Allogenic FMT using lean vegan donors in individuals with hepatic steatosis shows an effect on intestinal microbiota composition, which is associated with beneficial changes in plasma metabolites and markers of steatohepatitis.

## Abbreviations

NAFLD	Nonalcoholic fatty liver disease
MAFLD	Metabolic Associated Fatty Liver Disease
HCC	Hepatocarcinoma
FMT	fecal microbiota transplantation
NASH	nonalcoholic steatohepatitis
ALD	alcoholic liver disease
HE	hepatic encephalopathy
LPS	lipopolysaccharide
TLRs	toll-like receptors
SCFAs	short-chain fatty acids
BA	bile acids
FXR	farnesoid X receptor
TGR5	Takeda G-protein coupled receptor clone 5
ASBT	apical sodium dependent bile acid transporter
NF-κB	Nuclear factor-κB
HFD	high-fat diet
TLR4	toll-like receptor 4
US	ultrasound
ALT	Alanine aminotranferase
OCA	Obeticholic acid
NAS	NAFLD activity score
FGF-19	Fibroblast Growth Factor 19
BMI	Body Mass Index
AST	aspartate aminotransferase
GGT	gammaglutamyl transferase
LDL	low-density lipoprotein
HDL	high-density lipoprotein
IL	Interleukin
TNF-α	Tumor necrosis factor alpha
CK	Cytokeratin
TG	Triglycerides
FBS	Fasting blood sugar
TC	Total cholesterol
CFU	colony forming unit;
HOMA-IR	Homeostatic Model Assessment for Insulin Resistance
GLP-1	Glucagon-like peptide 1
FLI	Fatty liver index
NAS	NAFLD activity score
ELF	Enhanced liver fibrosis score
CRP	C-reactive protein
HbA1c	Glycated hemoglobin
LFC	Liver fat content
MRI	Magnetic resonance imaging
FIB-4	Fibrosis-4 Index for Liver Fibrosis;
